# Text mining of full-text journal articles combined with gene expression analysis reveals a relationship between sphingosine-1-phosphate and invasiveness of a glioblastoma cell line

**DOI:** 10.1186/1471-2105-7-373

**Published:** 2006-08-10

**Authors:** Jeyakumar Natarajan, Daniel Berrar, Werner Dubitzky, Catherine Hack, Yonghong Zhang, Catherine DeSesa, James R Van Brocklyn, Eric G Bremer

**Affiliations:** 1 School of Biomedical Sciences, University of Ulster at Coleraine, Cromore Road, Northern Ireland, UK; 2 Brain Tumor Research Program, Children's Memorial Research Center, 2300 Children's Plaza, M/C 226, Chicago, IL 60614, USA; 3 Division of Neuropathology, Department of Pathology, The Ohio State University, 4164 Graves Hall, 333 W. 10th Ave., Columbus, Ohio 43210, USA; 4 Windber Research Institute, Windber, PA, USA

## Abstract

**Background:**

Sphingosine 1-phosphate (S1P), a lysophospholipid, is involved in various cellular processes such as migration, proliferation, and survival. To date, the impact of S1P on human glioblastoma is not fully understood. Particularly, the concerted role played by matrix metalloproteinases (MMP) and S1P in aggressive tumor behavior and angiogenesis remains to be elucidated.

**Results:**

To gain new insights in the effect of S1P on angiogenesis and invasion of this type of malignant tumor, we used microarrays to investigate the gene expression in glioblastoma as a response to S1P administration *in vitro*. We compared the expression profiles for the same cell lines under the influence of epidermal growth factor (EGF), an important growth factor. We found a set of 72 genes that are significantly differentially expressed as a unique response to S1P. Based on the result of mining full-text articles from 20 scientific journals in the field of cancer research published over a period of five years, we inferred gene-gene interaction networks for these 72 differentially expressed genes. Among the generated networks, we identified a particularly interesting one. It describes a cascading event, triggered by S1P, leading to the transactivation of MMP-9 via neuregulin-1 (NRG-1), vascular endothelial growth factor (VEGF), and the urokinase-type plasminogen activator (uPA). This interaction network has the potential to shed new light on our understanding of the role played by MMP-9 in invasive glioblastomas.

**Conclusion:**

Automated extraction of information from biological literature promises to play an increasingly important role in biological knowledge discovery. This is particularly true for high-throughput approaches, such as microarrays, and for combining and integrating data from different sources. Text mining may hold the key to unraveling previously unknown relationships between biological entities and could develop into an indispensable instrument in the process of formulating novel and potentially promising hypotheses.

## Background

The platelet-derived lipid mediator sphingosine-1-phosphate (S1P) is an endogenous ligand of the endothelial differentiation gene (EDG) family of G protein-coupled receptors [1]. S1P is involved in various cellular responses such as apoptosis, proliferation, and cell migration [2,3]. The specific effects of S1P on glioblastoma cells have begun to be explored. S1P is mitogenic and stimulates motility and invasiveness of glioblastoma cell lines *in vitro *[4,5]. Moreover, high levels of expression of the enzyme that forms S1P, sphingosine kinase-1, correlate with shorter survival of glioblastoma patients [6]. However, the mechanisms behind the effects of S1P on glioblastoma cells *in vitro *and on the malignancy of glioblastomas *in vivo *remain largely undetermined.

Glioblastoma multiforme (GBM) is the most frequent and most malignant brain tumor accounting for approximately 12–15% of all intracranial neoplasms and 50–60% of all astrocytic tumors [7]. Glioblastomas are composed of poorly differentiated neoplastic astrocytes and affect predominantly adults [7]. The progression of glioma to malignant glioblastoma usually involves neovascularization [8]. We have investigated the roles played by S1P in regulating the malignant behavior of human gliomas. Using a panel of human glioma cell lines we determined that S1P was mitogenic for approximately 50% of the cell lines tested [4]. In addition, S1P stimulated motility and invasiveness through Matrigel of 60% of human glioma cell lines tested [5]. S1P is known to have different effects on cell migration depending upon which of its receptors are expressed. S1P signaling through S1P_1 _and S1P_3 _receptors enhances cell migration, while S1P_2 _signaling blocks migration [9]. Thus, whether a glioma cell line responds to S1P with proliferation or motility, or both or neither, is due to the profile of S1P receptor expression. The cell line used in this study, U-373 MG, expresses all three of these S1P receptors at similar levels and responds to S1P both mitogenically and with enhanced motility and invasiveness. Cell lines that do not respond mitogenically to S1P express extremely low levels of the receptor S1P_1 _[5], suggesting that this receptor is crucial for mediating S1P-stimulated glioma cell proliferation. Conversely, glioma cells in which S1P stimulates motility express high proportions of S1P_1 _and S1P_3_, relative to S1P_2 _[5]. By overexpressing or knocking down S1P receptor expression in glioma cells, Lepley *et al*. showed that the S1P_2 _receptor mediates inhibition of migration, while S1P_1 _mediates enhanced glioma cell migration in response to S1P [3]. Malchinkhuu *et al*. confirmed that S1P inhibits migration of some glioma cell lines through S1P_2 _signaling [10]. They also suggested that S1P_2 _is up-regulated in astrocytoma cells in comparison to normal astrocytes based upon receptor expression in glioma cell lines and GBM tissue [10]. However, their analysis of GBM tissue utilized only two cases. We recently examined expression levels of S1P_1_, S1P_2_, and S1P_3 _by real time PCR analysis in 48 cases of GBM in comparison to 20 cases of the relatively benign pilocytic astrocytoma [6]. We found no significant difference in expression of S1P_1_, S1P_2_, or S1P_3 _between these two tumor types. However, S1P_2 _expression in GBMs was consistently lower than that of S1P_1 _or S1P_3_. Thus, although its expression level is high in some long term glioma cell lines, S1P_2 _is not likely to be a dominant S1P receptor in gliomas *in vivo*. This suggests that the pro-migratory effect of S1P may be dominant in glioma cells *in vivo*.

To date, the impact of S1P on human glioblastoma is not fully understood. To gain new insights in the effects of S1P on this type of malignant tumor, we used gene expression analysis to investigate the response of a glioblastoma cell line (U373MG) to S1P administration in culture. Seventy-two genes were found to be differentially expressed (six genes are down-, whereas 66 are upregulated as response to S1P).

It has been estimated that only 20% of biological information and data are available in structured format or database systems. The remaining 80% are coded in natural language in technical reports, web sites, research publications and other text documents [11]. To elucidate the possible relationships and pathways of the genes that we found to be differentially expressed uniquely as a response to S1P, we have developed a system that facilitates the discovery of such relationships from the scientific literature. As manual information extraction (i.e., exhaustive reading of papers by humans) is very time-consuming and costly, many text mining and information extraction methods have been developed for automatic extraction of interaction and pathway information from the scientific literature [12,13]. By processing only the abstracts of papers, most of these systems were developed and tested on small portions of the available data. Some of the commercially available software packages such as Pathway Central^® ^(Ariadne Genomics, Inc.) are based on Medline abstracts.

Text mining of biomedical literature has already been applied successfully to various biological problems including the discovery and characterization of molecular interactions (protein-protein [14-18], gene-protein [12], gene-drug [19]), protein sorting [20,21], and molecular binding [22]). Most of these examples, however, have been based on the abstracts of research articles. The primary reason for this focus is the easy availability through MEDLINE and because they provide a highly concentrated source of information. There are currently more than 15 million abstracts in MEDLINE and more than 40 000 abstracts are added monthly. Full-text articles, on the other hand, are more comprehensive, more specific and detailed to address questions in biomedical research and development. Little research is available on text mining of full-text biological literature as opposed to article abstracts. The literature on mining of full-text documents in biology and medicine is much more limited than that on abstracts. For example, Shah *et al*. performed a systematic comparison of full-text and abstracts with respect to the information pertaining at keywords [23]. Shah *et al*. conclude that information extraction should be performed using full text articles. Yu *et al*. used full text articles to find synonyms of gene names that are not mentioned in abstracts [24]. Friedman *et al*. explored the distribution of molecular pathways in abstracts versus full text in single review papers [18]. Full-text documents often contain novel and important information not contained in the article's abstract [25]. Recently, Schuemie *et al*. applied information retrieval based approaches and compared the distribution of information in full-text versus abstracts [26]. The results of their study showed that the highest information *coverage *is located in the results section, while abstracts have the highest information *density*. Schuemie *et al*. argue for using full-text articles instead of abstracts.

This study presents an actual attempt to apply text mining in the context of a real biological research setting. The goal of this study is to decipher the impact of S1P on glioblastoma cell lines U373 MG. We are particularly interested in the effect of S1P on invasivity and downstream cascading events that could result from differential gene expression as a response to the stimulus. These events are described in gene-gene interaction networks, which we constructed based on pair-wise interaction patterns derived from text mining. Motivated by the results by Schuemie *et al*. [26], we decided to mine full-text biomedical articles. This study demonstrates that based on the text mining results involving full-texts of 20 peer-reviewed journals publishing cancer research papers, in combination with a novel approach for constructing interaction networks, it is possible to detect interesting gene relationships that might shed new light on our understanding of the cascading events triggered by S1P. Particularly, our study links S1P to the activation of MMP-9, a major culprit in tumor invasion. Matrix metalloproteinases (MMPs) are believed to play a crucial role in the malignant behavior of cancer cells such as rapid tumor growth, invasion, and metastasis by degrading extracellular matrix [27]. MMP-9 appears to be a key player in glioma invasion and angiogenesis [8], and has been shown to play an important role in aggressive behavior in a wide range of tumors [28].

## Results

### Extraction of gene relationships based on text mining

Motivated by the results from [23,24,26], we collected full-text articles published in 20 peer-reviewed journals in the field of molecular biology and biomedicine related to cancer research over a five-year period (1999 to 2003). The selection criteria of these journals were based on our research interest in brain tumors, journal impact factors, publisher representation and availability of electronic forms. The articles were downloaded from the journal sites using the automatic download agent *GetItRight *(CTH Technologies, Oakbrook, IL), as previously described [25]. All articles were downloaded as HTML text without images and then converted into XML documents. The resulting corpus of documents comprises 119 332 full-text articles. Table 1 provides a list of the selected journal articles and the total number of articles from each journal.

The interactions between the genes and proteins were extracted by text mining and natural language processing (NLP) methods as described in [25]. To summarize, our NLP methods comprise the following steps:

1. Tokenizing the text into sentences;

2. Parsing the sentences to identify noun phrases and verb phrases;

3. Selecting sentences that contain gene annotations using provided gene/protein name, relation, and synonym dictionaries;

4. Extracting gene annotations using pattern matching rules.

The text mining and further extraction of gene relations were performed using LexiQuest Mine^® ^(SPSS, Chicago, IL) with in-house developed dictionaries of gene/protein names, synonyms, and gene relationship information [25]. We performed a full-text parsing of individual article sections (abstracts, introduction, materials and methods, results and discussion, figure legends, and table captions) followed by extraction of pair-wise relationships between genes and proteins at a sentence level. For example, in the following sentence the gene/protein names and their relationship, identified by the verb, are flagged as follows.

Example 1:

Nevertheless, <*beta-catenin*> elicited a further <*increase*> in <*arf protein*> (2.5-fold after normalization for alpha-tubulin, figure b).

The extracted pattern from this sentence is hence 'beta-catenin | increases | arf protein.' Full pattern extraction was not possible for all sentences because the verb could not always be identified, as shown in the following example:

Example 2:

The extreme n terminus of aky2p has the ability to target cytoplasmic passengers, i.e. murine <*dihydrofolate reductase*> or <*ura6p*> from yeast, to mitochondrial locations ().

Here the extracted pattern is 'dihydrofolate reductase | NULL | ura6p', where NULL indicates that the type of relationship could not be specified ('NULL-pattern').

### Gene synonym dictionary

A major problem in the interpretation of the extracted patterns is the plethora of gene aliases. We created a synonymy dictionary with a preferred (or canonical) name for each gene or protein. This dictionary was compiled on the basis of Entrez Gene (formally LocusLink) as primary source and from other publicly available databases. The gene dictionary currently comprises 282 882 unique gene and protein names and 274 845 synonyms. We matched the extracted patterns against this dictionary and replaced each gene name by a canonical gene name to curate the extracted patterns. This functionality is realized via a *curator module *that replaces each gene and protein name by its canonical term. For instance, the curated pattern for Example 1 is 'CTNNB1 | increases | ARF', because CTNNB1 is the preferred expression for beta-catenin.

### Data warehousing

**Figure 1:**
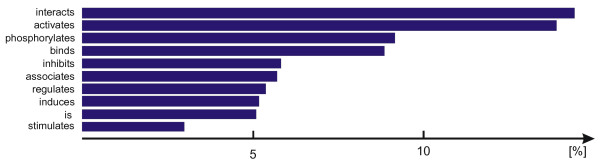
**Frequency distribution of relationship types**. Relationships were identified from sentence level extraction using LexiQuest Mine (SPSS, Chicago,IL) and patterns developed as previously described [25]. In total, 54 types of relationships were identified. The name and percentage occurrence of the top 10 relationships are shown. The most frequent type of relationship refers to 'interaction' (14.4%); the least frequent type refers to 'homo-oligomerization', featured in only two patterns.

We developed a data warehouse based on a relational database management system (RDBMS) to store the total of 455 222 patterns of extracted pair-wise interactions. The organization of the pre-processed information in the data warehouse facilitates efficient analysis and cross-referencing of the stored patterns with their source. The number of unique gene or protein names is 30 538 with TP53 accounting for the most frequent term that occurs in 16 431 patterns (3.6%). Among all patterns, 85 149 (18.7%) were complete, specifying both genes and the type of the relationship. The majority of the remaining relationships were missing the type of relationship. In total, 54 distinct types of relationships were identified. Figure 1 shows the distribution of the ten most frequent types of relationships.

**Figure 2:**
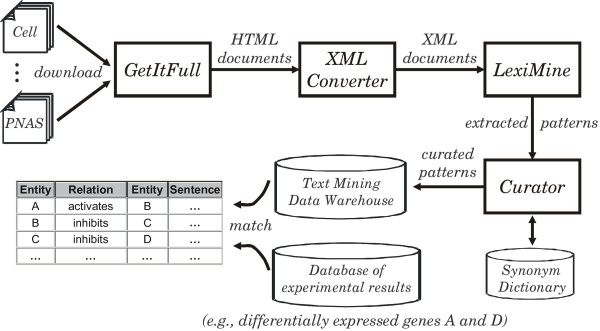
**Schematic diagram of the text mining analysis pipeline**. Full-text articles are downloaded and processed using the download agent *GetItRight*. The resulting HTML files are converted to XML. Biological entities (genes, proteins) and their relationships (activation, inhibition, etc.) are extracted from LexiQuest Mine (SPSS, Chicago, IL). The resulting patterns are stored in the text mining data warehouse. The text mining data is matched with results from a differential gene expression experiment.

The data warehouse also contains the full-text sentence from which the respective patterns were extracted. Figure 2 depicts a schematic summary of the pattern extraction process and the data warehouse.

### Deriving gene-gene interaction networks

One of the key results of gene expression studies based on microarrays is a list of genes that are differentially expressed under specific experimental conditions. Statistical methods are often used to identify these differently expressed genes. These methods, however, are unable to provide information on the biological implication or relationship among the genes on the list. The researcher often faces the tedious task of establishing functional relationships between the differentially expressed genes and analyzing potential downstream cascading events. To retrieve networks of interactions between the genes of interest, the data warehouse described above was matched against a table comprising results from a gene expression analysis after stimulation of a human glioma cell line (U373MG) with S1P. We excluded incomplete patterns ('NULL-patterns'), because they cannot be meaningfully included in the network generation process. Some of the NULL-patterns might describe interesting relationships and hence could be relevant for our research question. However, note that this does not affect the accuracy of the interaction networks that are derived from complete patterns.

The S1P gene list was derived from Affymetrix Gene Chip experiments. Differential gene expression was determined by comparison of resting U373MG cells with those stimulated with either S1P or EGF for 1 hour (see Methods). Similarly to S1P, EGF stimulates proliferation and motility/invasivity of cultured U373MG cells. In this experiment EGF stimulation served as a control to help identify differential gene expression due to common cellular processes. We identified 88 differentially expressed genes common to stimulation with either EGF or S1P. Many of these genes were related to the cell cycle suggesting a relationship to the proliferation phenotype. In total, 84 genes were identified as being unique to EGF stimulation. Seventy-two genes were differentially expressed specifically in response to S1P.

In the present study, we were interested in discovering interaction networks involving the set of 72 differentially expressed genes in response to S1P. Genes were considered differentially expressed if their *p*-value is smaller than 0.15. This relatively liberal choice for the cut-off relaxes the inclusion criterion for genes in the text mining analysis and is motivated by the assumption that even small changes in expression might be biologically relevant. We were particularly interested in the effect of S1P on invasivity. The S1P gene expression results can be thought of as two interaction networks: A network of interactions that links the differentially expressed genes to the stimulant, S1P, and another interaction network that links the gene list to the invasivity phenotype. The inclusion criteria to select relationships from the data warehouse for the former network were relationships that contained explicitly either 'S1P'or 'sphingosine-1-phosphate' in the sentence field. Similarly, the key words from the sentence field for the network linking the genes to invasion are: 'invasive', 'invasion', 'invasivity', and 'invasiveness'. Both of these networks were represented as directed pseudographs (see Methods).

### Gene interaction networks suggest S1P-mediated events leading to tumor invasivity

**Figure 3:**
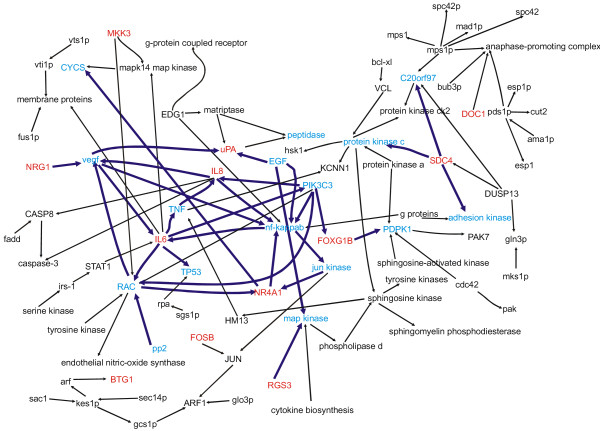
**S1P-Network**. Interaction network for differentially expressed genes (sentences related to S1P). The directed pseudograph of relationships related to S1P was generated as described in materials and methods. Seed vertices (shown in red) are the gene names from the list of 72 differentially expressed genes. Blue vertices and bold purple arcs represent genes and relationships that were found in this interaction network and in the interaction network related to invasivity shown in Figure 4.

**Figure 4:**
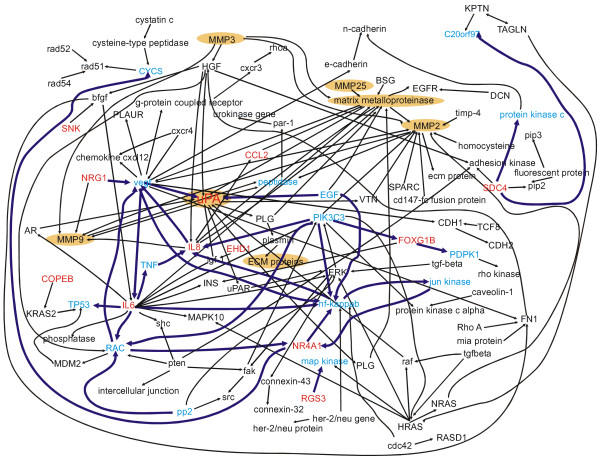
**nvasion-Network**. Gene interaction network of genes related to invasivity. The directed pseudograph of relationships related to invasivity was generated as described in materials and methods. Seed vertices (shown in red) are the gene names from the list of 72 differentially expressed genes. Blue vertices and bold purple arcs represent genes and relationships that were found in this interaction network and in the interaction network related to S1P shown in Figure 3. Genes directly related to matrix metalloproteinases (key components of invasivity) are highlighted by the mustard-colored ovals.

Figure 3 depicts an interaction network where the red vertices are the differentially expressed genes. A similar network related to invasion is shown in Figure 4. The blue vertices and purple arcs are those gene names and relationships contained in both networks and show an intersecting sub-network. All red vertices represent genes that are significantly up-regulated.

**Figure 5:**
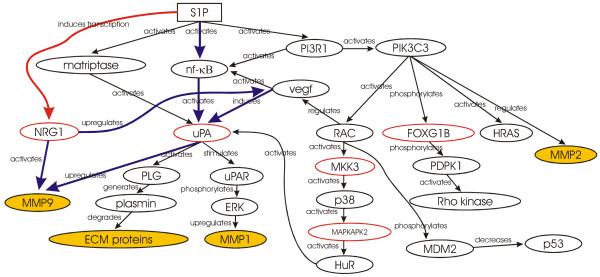
**Intersection-Network**. Gene interaction network derived from an intersection of the S1P- and invasion-network. This interaction network was hand drawn using gene names found in the S1P (Figure 3) and invasivity (Figure 4) networks as input vertices. In addition to the direction of the relationship shown by the arrow, the type is also depicted as text superimposed onto the arrow. The resulting graph contains several genes differentially expressed in the presence of S1P. These are shown in the red ovals. Genes directly related to matrix metalloproteinases are highlighted by the mustard-colored ovals. Key relationships describing the most direct connections between S1P and invasivity are highlighted by the bold purple arrows. The red arrow indicates that S1P induced transcription of NRG-1 in the microarray experiments.

We visually inspected both networks and verified the extracted relationships by checking the respective full-text sentences. As expected, the network in Figure 4 contains several MMPs and uPA, which are believed to play a pivotal role in tumor invasivity by degrading extracellular matrix [27]. Based on the blue vertices and purple arcs that defined an intersecting sub-network, we manually distilled those patterns that appeared to link S1P to invasivity. The resulting sub-network graph is depicted in Figure 5. One of the genes on the S1P list, uPA, stood out as an important hub in this sub-network. It could directly (MMP-9) and indirectly (MMP-1) activate MMPs as well as other extracellular matrix proteins. It could also be related directly back to S1P through the transcription factor NF-κB [19]. Figure 5 also shows an interesting link between S1P, neuregulin (NRG-1), and MMP-9. Our microarray data show that NRG-1 is upregulated by S1P in U-373 MG cells. In addition to being directly activated and upregulated by S1P, NRG-1 directly activates MMP-9 and indirectly activates uPA through up-regulation of vascular endothelial growth factor (VEGF). The specific relationship NRG-1 → MMP-9 was extracted in the present study from an article by Yao *et al*. [28]. This study revealed that NRG-1 activates MMP-9 via multiple signaling pathways (ERK-, PKC-, and p38 kinase-pathway) in human breast cancer cell lines [28].

In addition, the network in Figure 5 contains several additional known pathways leading to the activation of uPA and invasion. For instance, S1P is known to activate PI-3 kinase [29,30] in several cell types including glioma cells [4]. PI-3 kinase signaling through AKT and NF-κB is known to stimulate uPA expression and secretion [31].

### Comparison to other systems based on text mining

We compared the results of our study with three other systems that rely on text mining results of abstracts only: (1) iHop [32], (2) PathwayStudio Central^® ^with its proprietary ResNet^® ^database 3.0 [33], and (3) PubGene [34].

iHop (Information Hyperlinked over Proteins) is a free academic service that allows the user to retrieve sentences from PubMed abstracts that match a specified gene/protein name. iHop uses gene/protein names as hyperlinks between sentences in these abstracts, so that the abstracts in PubMed can be converted into one navigable resource. For a user-defined gene or protein name, iHop extracts one key sentence from each PubMed abstract that contains this specific name and a link to other genes or proteins. We retrieved the abstract sentences for NRG-1 and searched for sentences linking NRG-1 to either S1P or MMP-9 and found the publication by Atlas *et al*. [44], linking NRG-1 to up-regulation of MMP-9 in mouse breast cancer cells *in vivo*. We then retrieved all sentences related to the S1P receptors EDG-1, EDG-3, EDG-5, EDG-6, and EDG-8. We checked each sentence for the co-occurrence of the terms 'NRG-1', 'neuregulin-1', 'heregulin-β1' (synonym to NRG-1), and 'MMP-9'. The receptor EDG-8 was found in connection with the term 'NRG-1'. When we checked the associated abstracts, however, it became clear that this term refers to the G protein-coupled receptor EDG-8 (synonym to NRG-1 [35]) and not to neuregulin-1. In summary, we could not find any links between S1P or its EDG receptors and neuregulin-1. iHop provides a tool for constructing gene-gene interaction networks, but based on sentences that need to be selected manually. Thereby, it is possible to create an interaction network linking NRG-1, MMP-9, uPA, and VEGF; however, the user needs to sift through a large number of sentences to retrieve the corresponding information.

ResNet 3.0 contains molecular interactions for human, mouse, and rat, compiled on the basis of Medline abstracts. The current version ResNet 3.0 was released in August 2005, and the current number of Medline abstracts is approximately 15 million. To our knowledge, this represents one of the most exhaustive databases of scientific abstracts commercially available today. We used PathwayStudio Central and ResNet 3.0 to infer direct interaction networks between NRG-1 (or heregulin-β1), S1P receptors, uPA (a.k.a. PLAU), and MMP-9. For the interaction between NRG-1 and MMP-9, PathwayStudio Central depicts an inhibitory effect that is described by Puricelli *et al*. [36]. PathwayStudio Central also retrieves the publication by Yao *et al*. [28], which describes the activation of MMP-9 by NRG-1. PathwayStudio Central identifies a relationship between PLAU (uPA) and NRG-1 that is not contained in our data warehouse. This relationship was extracted from the following sentence: 'A specific antagonist of uPA receptor completely blocked the formation of these luminal glandular structures induced by PGE2 and HRG.' [abstract from [37]]. The activation of MMP-9 via PLAU (uPA) is extracted from [38-40]. Interestingly, the network produced by PathwayStudio Central retrieves a link between neuregulin-1 and EDG-5. The full sentence from which the relation is extracted is: 'Chromosomal mapping employing a rat somatic cell radiation hybrid panel demonstrated that nrg-1 is linked to marker D8Rat54 and tightly associated with H218 on chromosome 8.' In this context, 'nrg-1' refers to EDG-8, and H218 refers to EDG-5. However, the system assumes that NRG-1 represents EDG-8 in the interaction NRG-1 → EDG-5, but it assumes that NRG-1 represents neuregulin in the interaction NRG-1 → MMP-9. This problem is due to the polysemy of NRG-1.

Like iHop, PathwayStudio Central converts the term 'S1P' into membrane-bound transcription factor protease, site 1 (MBTPS1), since it is based on protein or gene names and not lipid molecules. As an alternative approach, we searched for interactions based on the S1P metabolic enzymes, sphingosine kinase (SPHK), sphingosine-1-phosphate lyase 1 (SGPL1) and sphingosine-1-phosphate phosphatase 1 (SGPP1) with NRG-1, MMP-9, NF-κB, and PLAU, but without any success.

PubGene comprises a database and analysis software for detecting relationships between genes and proteins, diseases, cell processes, cellular components, and drugs based on their statistical co-occurrence in the abstracts of scientific papers [34]. PubGene provides a network browser for visualizing gene-gene interactions. Like PathwayStudio Central, PubGene is based on protein or gene names and not lipid molecules; hence, it converts the term 'S1P' into membrane-bound transcription factor protease, site 1 (MBTPS1). We constructed a network for the S1P metabolic enzymes, sphingosine kinase (SPHK), sphingosine-1-phosphate lyase 1 (SGPL1) and sphingosine-1-phosphate phosphatase 1 (SGPP1) with NRG-1, MMP-9, NF-κB, and PLAU. In the resulting network, NRG-1 is linked to ERB2, ERB3, ERB4, EGFR, DSTN, and MMP-9. The connection to MMP-9 is described in a single publication that links up-regulation of neuregulin and MMP-9 in rat pancreatic carcinoma cell lines [41]. Importantly, PubGene is based on statistical co-occurrences of terms, which is not limited by sentence boundaries. In fact, the terms 'neuregulin' and 'MMP-9' occur in different sentences in the abstract from [41]. On the other hand, the arcs in the PubGene network do not indicate the type of the interaction; hence, the user needs to infer the particular relationship between NRG-1 and MMP-9 from the abstract. Interestingly, NRG-1 is not polysemic in PubGene as it refers unambiguously to neuregulin and not to EDG-8.

## Discussion

The overall utility of our text mining approach, including the strategy for constructing interaction networks, is demonstrated in the relationships discovered from the S1P gene list. Importantly, our text mining approach extracts and specifies the type of the interaction (e.g., 'activation', 'inhibition', etc.) explicitly. The experimental results indicate that addition of S1P induced overexpression of NRG-1 and uPA (and other genes) in a glioblastoma cell line and increased motility/invasivity. The relationship between NRG-1 and uPA leading to activation of MMP-9 was identified from both abstract-based text mining and our full-text based mining.

As shown in [26], omitting sections of text can entail a serious loss of information. Full-text, including figure and table captions, might be more appropriate than abstracts alone to infer patterns of pair-wise gene-gene interactions. On the other hand, mining full-text necessarily increases the noise, reflected by the huge number of NULL-patterns that we needed to exclude from further analysis. It is noteworthy that depending on the specific analysis task at hand, abstracts might be the better choice, as demonstrated in the study by Ehrler *et al*. who achieved a higher accuracy in text categorization by using abstracts only [42]. Gay *et al*. extracted key words for indexing from various sections of texts [43]. They obtained significantly better indexing results based on the sections results, results and discussion, conclusions, abstract and title, as compared to abstract and title alone. However, they also observed that the naïve use of complete manuscripts reduces the precision. Therefore, we cannot generally recommend the use of full-text articles instead of abstracts only.

The microarray data showed that NRG-1 is up-regulated by S1P in U-373 MG cells. The role of NRG-1 in tumor invasion and metastasis is still unclear [28]. A study by Yao *et al*. revealed that NRG-1 activates MMP-9 via multiple signaling pathways (ERK-, PKC-, and p38 kinase-pathway) in human breast cancer cell lines [28]. From the publication of Yao *et al*., the specific relationship NRG-1 → MMP-9 was extracted in the present study. Yao *et al*. proposed two models that might explain their observations. First, there is cross-talk between different signaling pathways and the blockage of one pathway leads to the activation of the other pathways. Alternatively, it might be possible that the transcriptional activation of MMP-9 requires the input of all three pathways. This input might be merged to a common target complex that must exceed a certain threshold value. This hypothesis explains why blockage of a single pathway can counteract activation of MMP-9, whereas increased signals from one single pathway can activate MMP-9. Yao *et al*. conclude that by blocking NRG-1, it is possible to inhibit MMP-9 activation and thereby inhibit cancer metastasis and angiogenesis. More recently, Atlas *et al*. have shown that in the mouse model, heregulin induces aggressive breast cancer behavior, via up-regulation of MMP-9 and VEGF [44]. NRG-1 upregulates VEGF in human breast cancer cell lines [45]. VEGF is one of the numerous proangiogenic molecules that have been identified to play an important role in the control of brain angiogenesis [46].

Many studies have implicated uPA in invasiveness of a variety of cancers including brain tumors [47,48]. It is tempting to hypothesize that in glioblastoma cell lines U373 MG, S1P induces invasion via cross-talk between pathways that include uPA, MMP-9, NRG-1, and VEGF. Figure 5 implies a multi-level regulation of uPA by S1P. S1P activates NF-κB to promote transcription of uPA [49]. Lysophosphatidic acid, which signals through receptors closely related to S1P receptors, is known to use this pathway to induce uPA transcription [50]. S1P activation of PI-3 kinase signaling through AKT and NF-κB is known to stimulate uPA expression and secretion [31]. Activation of Rac signaling through MKK3, p38 and MAPKAPK2 enhances stability of uPA mRNA [51]. MAPKAPK2 enhancement of uPA mRNA stability has been shown to be mediated by the RNA binding protein HuR [52]. S1P is well known to activate Rac [3,53,54] and p38 [55-57] in a variety of cell types. Further, it is known that S1P activates the serine protease matriptase [58], which has been shown to cleave and activate pro-uPA [59]. These data all suggest that S1P has the potential to effect transcription, message stability and activation of uPA.

We chose to extract gene-gene relationships from sentence-level linguistic processing. It is being debated as to what the best unit for text mining is. Advantages and disadvantages have been reported for all common text processing units including abstracts, sentences, and phrases. Ding *et al*. suggest that sophisticated text processing techniques are likely to be more beneficial to smaller text processing units because shorter lengths, simpler structures, and higher proximity of relevant verbs and biochemical nouns make their processing more tractable [60]. For example, appropriate verbs such as bind, inhibit, activate, in close proximity to biochemical terms are likely to be better indicators of an interaction than more distant verbs. Most of the patterns used in our linguistic extraction were designed with this in mind. The data warehouse developed for this study contains patterns in the form of: *gene A *| *interaction verb *| *gene B*.

The sentence-level processing approach, however, can produce the same pattern for very different statements. For example, both of the following sentences, *S*_1 _and *S*_2_, produce the same pattern *P*.

*S*_1_: 'It is highly questionable that gene A activates gene B'

⇒ *P*: *gene A *| *activates *| *gene B*

*S*_2_: 'It is highly likely that gene A activates gene B'

⇒ *P*: *gene A *| *activates *| *gene B*

To address this we manually checked the full-text sentences from which the patterns have been extracted and discarded those interactions for Figure 5 that are judged to be wrong or misleading. For instance, the interaction network in Figure 3 contains the relationship '*EDG1 | activates | nf-kappab*'; however, this pattern has been extracted from the following sentence: "The inability of <*edg-1*> to <*activate*> <*nf-kappab*> regardless of s1p cannot be attributed to low expression levels of the receptor, because edg-1 was expressed to a greater extent than both edg-3 and edg-5." Currently, we do not see any way how to solve this problem automatically. Future research will have to address this issue.

In order to meaningfully visualize the interaction networks, we developed a pruning strategy for selecting higher-level transitive dependencies that meet certain inclusion criteria (see Methods). All red vertices represent significantly up-regulated genes as response to S1P, which facilitates the interpretation of cascading downstream events in this study. Future work will need to focus on how to interpret the complex interplay between up- *and *downregulated genes in interaction networks.

A major problem in text mining of biological literature is polysemy, where the same abbreviation or name can refer to different biological entities. This problem has been recently reviewed [61]. In the S1P example described in the results section, the term NRG-1 can refer to neuregulin-1 or EDG-8, which has led to misleading results (and also caused some confusion to the authors). The synonym dictionaries that were developed for the present study also contain ambiguities. For example, 'Hsp90' is an alias for 'Hsp86' (heat shock 90 kDa protein 1, alpha) and 'HSPCB' (heat shock 90 kDa protein 1, beta). The name 'AR' is an alias for 'AREG' (amphiregulin, schwannoma-derived growth factor) and 'AkR1B1' (aldo-keto reductase family 1, member B1). Such examples of ambiguity can also be found in other systems, e.g., iHop. Case-sensitivity is another problem in our dictionaries. For instance, the canonical expression for 'Acc' is 'Acc', whereas the canonical term for 'ACC' is 'ACACA'. Although 'Acc', ACC', and 'ACACA' are synonyms for acetyl-Coenzyme A carboxylase alpha, there is a potential pitfall. If a pattern contains the expression 'Acc', then the curator module identifies it as canonical term and does not replace it. On the other hand, 'ACC' is replaced by 'ACACA'. In gene symbol naming conventions, it is accepted that the case does matter [62]. For example, 'PSA' refers to 'prostate specific antigen', whereas 'psa' refers to 'pleiomorphic adenoma gene 1'. Statistics about the problem of case-sensitivity in this context can be found in [62]. We consider gene name ambiguity as one of the major pitfalls in text mining of biological texts. Chen *et al*. recently invited the community to use only official symbols in their publications and to revise naming conventions [61], two essential goals that we believe need to be achieved in order to exploit the full potential of text mining. Future research will need to focus on *in silico *approaches to tackle this ambiguity problem. Intelligent text mining tools are needed to understand in which context NRG-1 refers to neuregulin-1 and in which context it refers to EDG-8.

Recent advances in the areas of genomics and proteomics have become increasingly dependent on high throughput approaches. Analysis and data mining of these experiments yield lists of genes or proteins that may not have a readily apparent relationship. The research literature is an obvious source to help uncover these relationships. Abstracts (e.g., MEDLINE) and full-text articles are two main sources of textual data in biology and biomedicine. The processing and analysis of full-text is more demanding and complex than mining abstracts only. First, it is computationally expensive. Second, the access to full-text documents can be limiting. Third, the more complex language structures make extraction of relationships more difficult. The approach described here and elsewhere [25,63] automates the process of downloading articles and concept extraction. Automation is a distinct advantage in the ability to update and maintain the data warehouse. With more and more articles becoming available electronically through open access publishing and library subscriptions it is becoming easier to obtain full-text articles.

## Conclusion

The famous quote by the biochemist Frank Westheimer, 'A couple of months in the laboratory can frequently save a couple of hours in the library', is more than ever relevant in modern research practice. In the present study, we demonstrated how text mining could be a potential addition to the toolbox helping to generate novel and promising hypotheses. We found that regulation of uPA, NRG-1 and MMP-9 by S1P could be a key player in the invasion of glioblastoma cells. Our methodology could be applied in similar studies investigating gene-gene relationships in high-throughput transcriptomic research. The results of the present study indicate that full-text articles from just a few years of a limited number of journals can provide sufficient information to obtain meaningful gene-gene relationships. However, is mining full-text to be preferred over mining abstracts only? We believe that this question cannot be answered in general but depends on the specific study design and, of course, on the available computational resources. Much more research is necessary on how to most effectively mine full-text articles and how to efficiently generate and visualize interaction networks. Key problems that need to be solved are ambiguities due to gene name polysemy, and modalities and negations, which can only be resolved by including contextual information.

## Methods

### Cell culture preparation

The human glioma cell line U373 MG (American Type Culture Collection, Rockville, MD, U.S.A.) were maintained in Eagle's minimum essential medium containing 10% fetal bovine serum (FBS), non-essential amino acids and sodium pyruvate (all media from Mediatech, Herndon, VA). Cells were grown at 37°C in 95% air and 5% CO_2_. Cultures were passaged once per week at a ratio of 1:12.

### Probe preparation

U-373 MG cells were treated for 1 hour with 100 nM S1P, 10 nM EGF or vehicle. RNA was extracted using Trizol (Invitrogen) according to manufacturer's instructions. First and second strand cDNA was synthesized using Superscript II reverse transcriptase and DNA polymerase I, and cDNA was purified using phase lock gel (Eppendorf). Synthesis and biotinylation of cRNA and hybridization were performed using the Enzo Bioarray High Yield RNA Transcript Labeling Kit in accordance with the manufacturer's instructions (Affymetrix, Santa Clara, California, USA). Biotinylated cRNA was then purified using the RNeasy MiniKit (Qiagen) and a sample was separated on a 1% agarose formaldehyde gel to verify RNA integrity.

An overnight ethanol precipitation was performed and cRNA was resuspended in 15 μl of DEPC treated water (Ambion, Inc.). cRNA was quantified. 20 μg of unadjusted cRNA was fragmented according to Affymetrix's instructions. The 5× fragmentation buffer included 200 mM Tris-acetate, pH 8.1, 500 mM KOAc, 150 mM MgOAc.

### Hybridization

Quantification of cRNA was adjusted from total RNA to reflect carryover of unlabeled total RNA with an equation given by Affymetrix. Added 15 μg of adjusted fragmented cRNA to a 300 μl volume hybridization cocktail which included final concentrations of 0.1 mg/mL of herring sperm DNA, 0.5 mg/mL acetylated BSA, and 1× MES hybridization buffer. The cocktail also contained hybridization controls: 50 pM of oligonucleotide B2 (Genset Corp.) and 1.5, 5, 25, and 100 pM of cRNA BioB, BioC, BioD, and Cre, respectively (ATCC). We hybridized 200 μl of the target to the Human Genome HuGeneFL microarray chip for 16 hours, according to Affymetrix's procedures.

### Washing, staining, and scanning

We washed the probe arrays with stringent (100 mM MES, 0.1 M [Na^+^], 0.01% Tween 20) and non-stringent (6× SSPE, 0.01% Tween 20, 0.005% Antifoam) buffers in the Affymetrix GeneChip Fluidics Station using pre-programmed Affymetrix protocols. We stained the probe arrays with streptavidin phycoerythrin (SAPE) and amplified the signal using biotinylated antibody solution. The SAPE stain contained 2× stain buffer (final 1× concentration: 100 mM MES, 1 M [Na^+^], 0.05% Tween 20, 0.005% Antifoam), 2 μg/μL acetylated BSA, and 10 μg/mL SAPE (Molecular Probes). The antibody amplification solution contained 2× stain buffer, 2 mg/mL acetylated BSA, 0.1 mg/mL normal goat IgG, and 3 g/mL biotinylated antibody. Stained in the GeneChip Fluidics Station using pre-programmed Affymetrix protocols. Scanned the probe arrays in the Affymetrix GeneChip Scanner.

### Microarray data analysis

The fluorescent intensity data from Affymetrix Microarray Suite Version 5 (MAS 5.0) were exported as CEL files and imported into Probe Profiler (Corimbia, Berkley, CA), which uses a model-based approach for statistical analysis of expression data.

Before any comparison, low or non-expressed genes, as determined by a minimum expression value cut-off, were excluded from any further analysis. The minimum expression cut-off value was determined from the *p*-value for expression. At an expression value of 50 the *p-*value for expression was greater than 0.05 for almost all genes indicating that expression values less than 50 could not be reliably distinguished from background. Thus, 50 was chosen as the minimal value for expression. Three cell lines stimulated by S1P and three unstimulated cell lines were investigated. One microarray experiment was carried out for each cell line and each condition. The expression values of the three microarrays per condition were averaged. Using the Probe Profiler software and unstimulated cells as a baseline, the mean (*n *= 3) gene expression values were used to identify transcripts significantly increased or decreased in U373MG cells stimulated with either EGF or S1P. In this comparison genes with a *p*-value (based on *t*-test) of less than or equal to 0.15 were considered to be differentially expressed.

### Network construction

**Figure 6:**
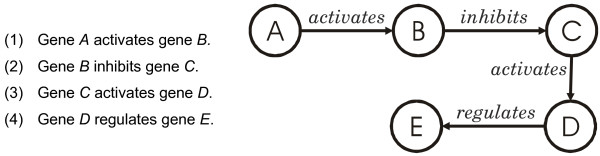
Example of transitive dependencies.

Assume that the database of experimental results contains only the gene *A*, and the data warehouse contains the following patterns that can be graphically represented as a directed graph (Figure 6):

In this example, gene *A *is involved in the inhibition of gene *C *via activation of gene *B*. Higher-order transitive dependencies include the regulation of gene *E *via the pathway involving *B*, *C*, and *D*. Informally, we can refer to the interaction *A *→ *B *as *direct interaction*, whereas *A *→ *B *→ *C *→ *D *→ *E *represents a *transitive dependency of degree 4*, because this dependency involves a path length of 4.

Informally speaking, a *graph *is a set of *nodes *(or *vertices*) that are connected by *links *(or *edges*). A *multigraph *is defined as a set *V *of vertices, a set *E *of edges, and a function *f*: *E *→ {{*u*, *v*}|{*u*, *v*} ∈ *V*, *u *≠ *v*}, specifying which vertices are connected by which edge. If *u *= *v*, then the graph is considered a *pseudograph*, i.e. it contains a loop connecting a vertex with itself. If the edges have a direction then the graph is referred to as *directed graph *or *digraph*. In the network structure in the present study the genes/proteins are represented as vertices and the relationships as directed edges. The network is a *directed pseudograph*, because it may contain multiple edges and loops between the same vertices.

**Definition 1**: *Transitive Dependencies of Degree 1*

Let *v*_*i *_denote a vertex (i.e., a gene/protein) and *e*_*ij *_denote an edge between *v*_*i *_and *v*_*j*_. Let *W *denote the set of all *n *patterns in the data warehouse, *W *= {{*v*_*i*_, *e*_*ij*_, *v*_*j*_}}; | *i*,*j *= 1..*n*}. Let *V*_1 _be the set of vertices that represent canonical gene names contained in the database of experimental results, *V*_1 _= {{*v*_1*i*_} | *i *= 1..|*V*_1_|}. The set of patterns that contain a vertex from *V*_1 _is *P *= {{*v*_*i*_, *e*_*ij*_, *v*_*j*_}} | *v*_*i *_∈ *V*_1 _∨ *v*_*j *_∈ *V*_1_}. Then the edges in *P *are defined as *transitive dependencies of degree 1 *or as *direct dependencies*.

**Definition 2**: *Transitive Dependencies of Degree 2*

Let *V*_2 _= {{*v*_2*i*_} | *i *= 1..|*V*_2_|, *v*_2*i *_∧ *P *∧ *v*_2*i *_∧ *V*_1_} and *Q *= {{*v*_*i*_, *e*_*ij*_, *v*_*j*_}} | {*v*_*i*_, *e*_*ij*_, *v*_*j*_} ∈ *W*, (*v*_*i *_∈ *V*_2 _∧ *v*_*j *_∧ *V*_1_) ∨ (*v*_*j *_∧ *V*_1 _∧ *v*_*j *_∈ *V*_2_)}. Then the edges in *Q *are defined as *transitive dependencies of degree 2*.

**Definition 3**: *Transitive Dependencies of Degree 3*

Let *V*_3 _= {{*v*_3*i*_} | *i *= 1..|*V*_3_|, *v*_3*i *_∈ *Q *∧ *v*_3*i *_∉ *V*_2_} and *R *= {{*v*_*i*_, *e*_*ij*_, *v*_*j*_}} | {*v*_*i*_, *e*_*ij*_, *v*_*j*_} ∈ *W*, (*v*_*i *_∈ *V*_3 _∧ *v*_*j *_∉ *V*_1 _∪ *V*_2_) ∨ (*v*_*j *_∉ *V*_1 _∪ *V*_2 _∧ *v*_*j *_∈ *V*_3_)}. Then the edges in *R *are defined as *transitive dependencies of degree 3*.

**Figure 7:**
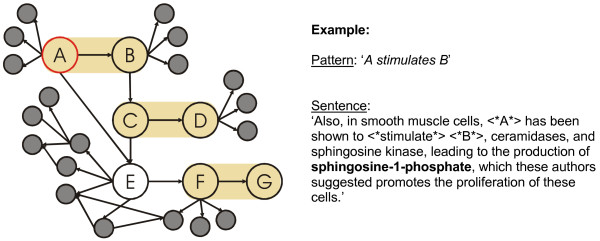
Example of interaction network.

**Figure 8:**
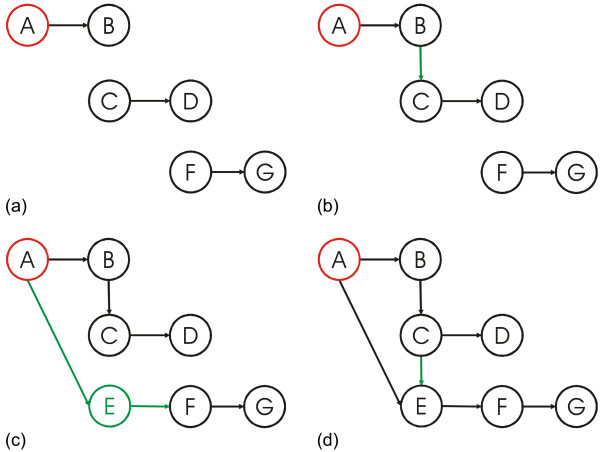
Pruning strategy for network construction.

Our previous empirical results have shown that the inference of transitive dependencies of *degree *> 3 are computationally very expensive. Furthermore, it is nearly impossible to visually inspect the inferred networks of higher-order degrees. For example, for the 72 differentially expressed genes, the data warehouse contains 418 unique direct interactions with other genes, which in turn are in a relationship with 21 882 other genes. For the latter, there exist 30 995 unique patterns of interaction with other genes, so that the network of transitive dependencies of degree 1, 2, and 3 would comprise a total of 53 295 vertices. Although it is possible to retrieve transitive dependencies of higher-order from the data warehouse, the resulting networks cannot be meaningfully visualized, which makes the interpretation of the extracted patterns both difficult and time-consuming. More importantly, many selected transitive dependencies may be irrelevant for the phenomenon under investigation. Therefore, we decided to implement a 'pruning' strategy as follows (illustrated in Figure 7 and 8):

1. Retrieve all patterns that specify transitive dependencies of degree 1, 2, and 3 for the set of differentially expressed genes.

2. Retrieve the sentences from which the patterns in (1) have been extracted.

3. Based on these sentences, identify all interactions that meet a specific inclusion criterion (an example for such a criterion is given below).

4. Retain only those patterns that meet the inclusion criterion.

5. Each pattern from (4) contains a pair of entities (i.e., canonical gene/protein names). Use each entity as *seed vertex *in the network.

6. For each seed vertex, find all transitive dependencies of degree 1, 2, and 3 that lead back to a differentially expressed gene. If a link exists between vertices that are involved in this path, then connect the vertices accordingly.

7. Find and display all interactions between the vertices from (6).

In the present study, we are interested in two types of networks: The network that links the differentially expressed genes to S1P and the network that links the genes to tumor invasivity. Hence, the inclusion criterion for the former network is that the sentences contain explicitly either 'S1P'or 'sphingosine-1-phosphate'. The key words for the network linking the genes to invasion are: 'invasive', 'invasion', 'invasivity', and 'invasiveness'. For visualizing the networks, we used the program *Pajek *[64].

The following figure illustrates the pruning strategy for the network linking the genes to S1P. In this example, assume that only gene *A *is differentially expressed. Figure 4 depicts a contrived network of interactions. Note that although multiple transitive dependencies of degree 1, 2, and 3 can be retrieved from the data warehouse, a visual representation as shown in this example is not possible due to complexity.

The highlighted patterns in Figure 7 are extracted from sentences containing one of the key words of the inclusion criterion. Many transitive dependencies comprise patterns that do not meet the inclusion criterion; these are represented by the grey vertices.

The vertices *A*, *B*, *C*, *D*, *F *and *G *are the seed vertices of the network as shown in Figure 8a. For each seed vertex, we find the transitive dependencies of degree 2 that lead back to *A *(Figure 8b, *B *→ *C*). Then, for each seed vertex we find the transitive dependencies of degree 3 that lead back to *A *(Figure 8c, *A *→ *E *→ *F*). Finally, we retrieve all remaining interactions between the vertices (Figure 8d, *C *→ *E*). The network in Figure 8d is the resulting 'pruned' network.

## Authors' contributions

JN performed the text mining analysis and co-implemented the data warehouse and interaction networks with DB. WD and CH oversaw the design of the study and contributed significantly to writing the paper. YZ was involved in the gene expression experiments and analysis of gene expression data. He also was involved in the processing of articles and design of the data warehouse. CD was involved in the development of the text mining process and involved in the processing of articles. JRVB designed and conducted the experiments involving S1P stimulation of glioma cells. EGB oversaw the gene expression experiments and developed the text mining process described here.

## Figures and Tables

**Table 1:** Journals and number of downloaded articles.

	**Journal Name**	**# of Articles**
1	*Journal of Biological Chemistry*	24 846
2	*Science*	11 440
3	*PNAS*	11 345
4	*Journal of Neuroscience*	10 758
5	*Biochemical and Biophysical Research Communications*	7 529
6	*Biochemistry*	6 205
7	*Nature*	5 825
8	*FEBS Letters*	4 725
9	*Brain Research*	4 648
10	*Neurology*	4 612
11	*Cancer Research*	4 550
12	*Oncogene*	3 910
13	*Nucleic Acid Research*	3 492
14	*Cancer*	3 059
15	*International Journal of Cancer*	2 906
16	*EMBO Journal*	2 729
17	*Journal of Cell Biology*	2 625
18	*Neuron*	1 573
19	*Cell*	1 553
20	*Genes and Development*	1 002
	TOTAL	119 332
